# Psychiatry E-Learning for Foundation Doctors: Creation and Review of Four Modules

**DOI:** 10.1192/bjo.2025.10271

**Published:** 2025-06-20

**Authors:** Lauren Fitzmaurice, Felicity Allman, Prathiba Rao, Arthita Das

**Affiliations:** 1 Sheffield Health and Social Care NHS Foundation Trust, Sheffield, United Kingdom; 2 Cumbria, Northumberland, Tyne and Wear NHS Foundation Trust, Northumberland, United Kingdom; 3 Rotherham, Doncaster and South Humber NHS Foundation Trust, Rotherham, United Kingdom

## Abstract

**Aims:** Foundation doctors rotate through six specialties during their programme. These may/may not include a psychiatry placement. E-learning for health (E-lfh) is a free resource which maps to the professional capabilities in the foundation curriculum, including mental health capabilities. During a six month fellowship, 4 topics were either newly created or improved within the psychiatry e-learning: anxiety disorders, substance use disorder, self-harm assessment and management and medically unexplained symptoms. Following this, the plan was to assess the impact and effectiveness of the e-learning.

**Methods:** The selection of modules were based on requirements from E-lfh and collaboration with the Royal College of Psychiatrists. It was agreed that the modules should be designed with as much interactivity as possible for an e-learning package, aimed at a foundation doctor (not what should be expected from a psychiatry trainee or higher) and also to equip a doctor with fundamental psychiatric knowledge regardless of if they choose psychiatry as a career.

Two modules were redesigns of pre-existing modules – self harm and substance use disorder. These originally were four distinct modules (two for each of the topics). Therefore the learning for each module was redesigned and updated. Medically unexplained symptoms (MUS) and anxiety disorders were new modules.

Feedback has been obtained via the E-lfh website which collates feedback at the end of each module and scores content, presentation, interactivity, self-assessments and overall rating. A separate survey has also questioned foundation doctors in the Northern deanery about their accessing of e-learning and evaluation.

**Results:** On the E-lfh website, all 4 modules have been accessed with number of feedback left ranging from 3 (MUS) to 11 participants (substance use disorders). The scores rated content, presentation, interactivity, self-assessments and overall rating. All of which were rated 4.4/5 and above.

In the Northern deanery survey, out of 27 participants, only 1 had accessed the modules – MUS. The doctor had rated the session’s overall, clarity and relevance as good, with interactivity and engagement as average. They noted the difficulty as easy and rated their preparedness for psychiatry related cases as “somewhat prepared”.

**Conclusion:** Whilst the scores from the E-lfh portal suggest good feedback for the completed modules, the more local feedback suggests limited uptake for e-learning modules in general. Therefore, the next stage of the project will be to design focus groups to further elicit views of foundation doctors before a full report is generated with suggestions to improve uptake and accessibility.

